# High-throughput combination assay for studying biofilm formation of uropathogenic *Escherichia coli*

**DOI:** 10.1007/s00203-024-04029-w

**Published:** 2024-07-05

**Authors:** M. Li, C. D. Cruz, P. Ilina, P. Tammela

**Affiliations:** Drug Research Program, Division of Pharmaceutical Biosciences, Faculty of Pharmacy, University of Helsinki, P.O. Box 56, Helsinki, FI-00014 Finland

**Keywords:** UPEC, Resazurin, Crystal violet, Assay optimization, Biofilm, Screening

## Abstract

Uropathogenic *Escherichia coli*, the most common cause for urinary tract infections, forms biofilm enhancing its antibiotic resistance. To assess the effects of compounds on biofilm formation of uropathogenic *Escherichia coli* UMN026 strain, a high-throughput combination assay using resazurin followed by crystal violet staining was optimized for 384-well microplate. Optimized assay parameters included, for example, resazurin and crystal violet concentrations, and incubation time for readouts. For the assay validation, quality parameters Z′ factor, coefficient of variation, signal-to-noise, and signal-to-background were calculated. Microplate uniformity, signal variability, edge well effects, and fold shift were also assessed. Finally, a screening with known antibacterial compounds was conducted to evaluate the assay performance. The best conditions found were achieved by using 12 µg/mL resazurin for 150 min and 0.023% crystal violet. This assay was able to detect compounds displaying antibiofilm activity against UMN026 strain at sub-inhibitory concentrations, in terms of metabolic activity and/or biomass.

**Graphical Abstract:**

**Figure:**
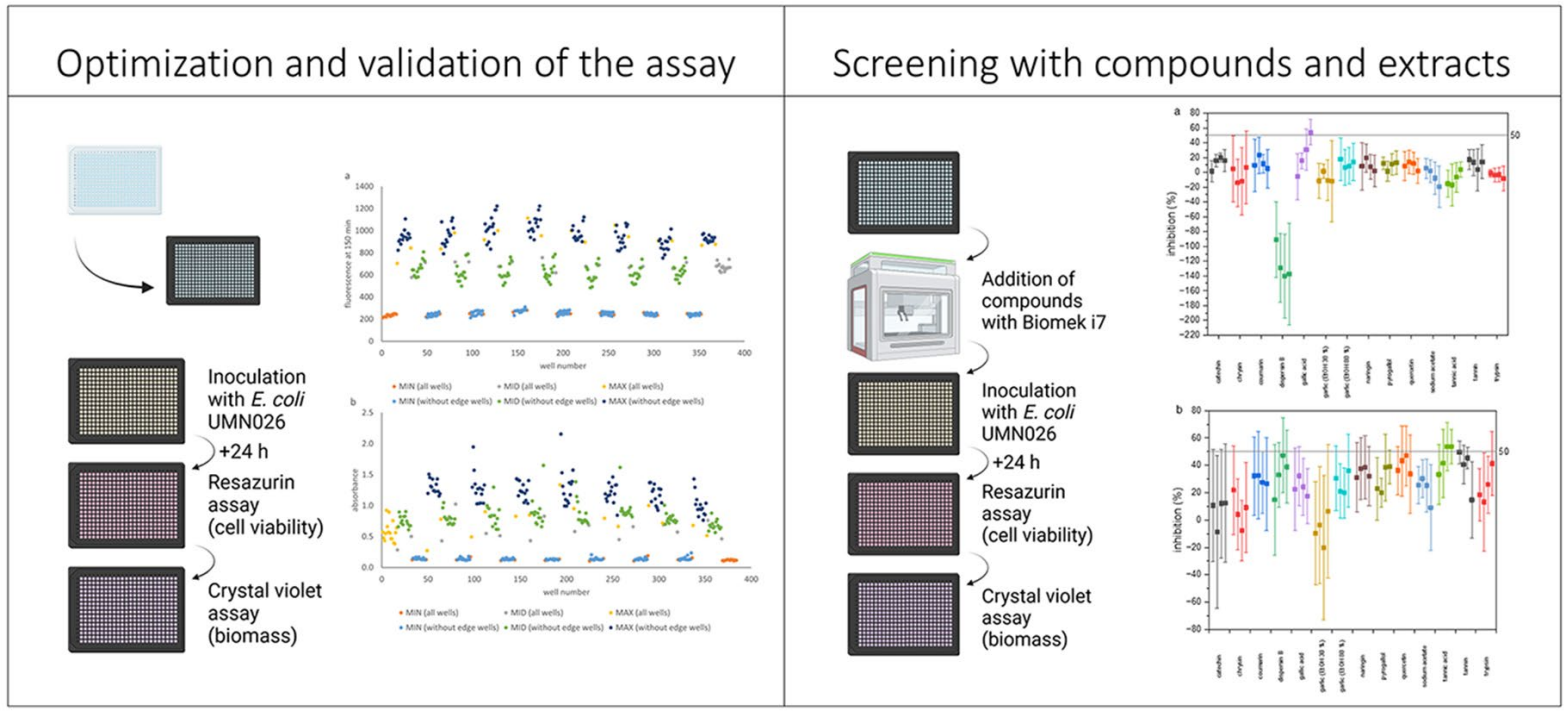

**Supplementary Information:**

The online version contains supplementary material available at 10.1007/s00203-024-04029-w.

## Introduction

Urinary tract infection (UTI) is regarded as the most common bacterial infection type that occurs in humans (Foxman 2003). Uropathogenic *Escherichia coli* (UPEC) is the primary pathogen causing hospital-acquired UTIs (Flores-Mireles et al. 2015). The formation of biofilm is a key virulence property of UPEC, which causes bacteria to be more resistant to antibiotics, in comparison to non-biofilm producing UPEC strains (Mittal et al. 2015). Biofilms are bacterial communities enclosed within a self-secreted extracellular matrix that is primarily comprised of exopolysaccharides, proteins, nucleic acids, and lipids (Karygianni et al. 2020). Biofilms exist in multiple forms depending on the growth platform (Branda et al. 2005). Compared to planktonic bacteria, microbes associated with biofilms generally possess enhanced capabilities to withstand antibiotic treatments (Fux et al. 2005). For example, it has been found that uropathogenic *Pseudomonas aeruginosa*, when inside a biofilm matrix, can withstand an antibiotic concentration that is 2500-fold of the minimum inhibitory concentration (MIC) used against planktonic cells (Nickel et al. 1985). In the course of infection, UPEC can form biofilms on abiotic surfaces, such as urinary catheters, or on urothelium surface. Adherent UPEC can invade bladder epithelium and form biofilm-like intracellular bacterial communities associated with recurrent UTIs (Delcaru et al. 2016). The complete eradication of biofilm-related infections is challenging given the absence of specific drugs.

Antimicrobial therapies are necessary for symptomatic UTIs, whose incidence is high (Foxman 2003). In addition, many UPEC strains are multidrug-resistant (MDR), due to common prescription of antibiotics, such as ciprofloxacin, to treat UTIs, and also the prophylactic use of antibiotics (Kot 2019; Raeispour and Ranjbar 2018). Due to the emergence of these MDR strains, there is an increased demand for new antibiotic treatments. Hence, it is important to develop and improve in vitro assays for screening of potential therapeutics, which can accelerate antibiofilm drug discovery. One of the options is to use high-throughput screening (HTS), in which a large library of compounds can be tested for potential effects against the target bacterial strain.

With that in mind, the aim of this study was to optimize an HTS method in a 384-well microplate format, for assessing the effects of compounds on the biofilm formation of UPEC. Since UPEC grows rapidly during an active infection (Forsyth et al. 2018), using urine as a source of nutrients (Reitzer and Zimmern 2019), artificial urine (AU) was used as the growth medium for the assays in this work, to better mimic the in vivo growth environment. Two assays were performed in sequence using the same microplate: a resazurin-based assay to measure cell viability followed by a crystal violet staining assay for biomass quantification. Both assays have been extensively used to study biofilm of bacterial cells (Paytubi et al. 2017), and it is known that they can be used as a combination assay in the same microplate for several bacterial species (Gilbert-Girard et al. 2020; Paytubi et al. 2017; Skogman et al. 2012). As the ability to form biofilm is affected by multiple factors, the assay conditions have to be verified for each specific assay medium-strain combination. To our knowledge, this is the first study utilizing a combination HTS 384-well microplate-based assay to evaluate the biofilm of UPEC.

The optimized assay was validated according to the guidelines described by Iversen et al. (2012). Screening of a set of compounds and a garlic extract, potentially active against UPEC biofilms was done to evaluate the performance of the assay.

## Materials and methods

### Materials

Prior to the combination assays, preliminary assays were performed using clear 384-well microplates (Nunc™ 242757, Thermo Fisher Scientific, Roskilde, Denmark). Further details can be found in the supplementary document. Subsequently, combination assays were performed in black 348-well microplates with a transparent flat bottom (pureGrade™ S, BRAND, Wertheim, Germany).

AU was prepared as described by Eberly et al. (2017). It was stored at 2–8 °C and warmed to 37 °C prior to use. Resazurin solutions were prepared from resazurin sodium salt (Sigma-Aldrich, St. Louis, MO, USA) and sterilized by filtration using a 0.2 μm cellulose acetate syringe filter (VWR, Radnor, PA, USA). Sterile phosphate buffered saline (PBS) solution was used to dilute resazurin for the assays. Crystal violet solutions were prepared by diluting 1% crystal violet (Sigma-Aldrich, St. Louis, MO, USA) with Milli-Q water.

Compound and garlic extract stocks were prepared from powder and dissolved into either 100% dimethyl sulfoxide (DMSO) (sterile-filtered; VWR, Leuven, Belgium) or sterile Milli-Q water. In three independent assays, newly prepared working solutions were used, diluted from the same stocks on the day prior to the assay, except for trypsin, whose new stocks were prepared each time due to limited stability.

Ethanolic garlic (*Allium sativum*) extracts (EtOH 30%, EtOH 80%) were prepared by Dr. Karmen Kapp and dispersin B by Honey Bokharaie (University of Helsinki, Faculty of Pharmacy). Other compounds were obtained from commercial sources: (+/-)-catechin (Sigma, St. Louis, MO, USA), chrysin (Extrasynthese, Genay, France), coumarin (Merck, Darmstadt, Germany), gallic acid (Sigma, St. Louis, MO, USA), naringin (Carl Roth, Karlsruhe, Germany), pyrogallol (Riedel-de Haën, Sigma-Aldrich, Seelze, Germany), quercetin crystalized (Merck, Darmstadt, Germany), sodium acetate (Sigma-Aldrich, St. Louis, MO, USA), tannic acid (Sigma, St. Louis, MO, USA), tannin (Extrasynthese, Genay, France), trypsin 1:250 (from porcine pancreas, PanReac AppliChem, Darmstadt, Germany), and tetracycline hydrochloride (Calbiochem, EMD Biosciences, La Jolla, CA, USA).

### Bacterial strain and growth conditions

The UPEC strain used was *E. coli* UMN026 (ATCC BAA1161, Microbiologics Inc., Saint Cloud, MN, USA), serotype O17:K52:H18, which is an MDR strain isolated from a female US patient, who had uncomplicated acute cystitis (Lescat et al. 2009; Mukherjee et al. 2023).

*E. coli* UMN026 was inoculated from a long-term stock stored at -80 °C to a lysogeny broth (LB) agar plate, grown at 37 °C overnight and stored at + 4 °C (monthly working culture). A weekly culture was inoculated from the monthly culture. On the first day of each assay, an overnight bacterial plate was prepared from the weekly culture. On the next day, a bacterial suspension was prepared in 0.9% saline solution, and diluted to 10^6^ colony-forming unit (CFU)/mL in AU for the inoculation of the 384-well microplate. The microplate was then incubated for 24 h at 37 °C without shaking, to ensure the formation of mature biofilm. The final volume in each well was 50 µL.

### Combination assay for biofilm assessment

#### Optimization of resazurin and crystal violet concentrations

The combination assay, in essence, means that the resazurin assay was performed first, followed by the crystal violet assay, in the same microplate. Three resazurin concentrations, 4 µg/mL, 12 µg/mL, and 24 µg/mL, were tested in combination with three crystal violet concentrations, 0.01%, 0.023%, and 0.1%. Both minimum signal wells (AU only), and maximum signal wells (untreated bacterial growth) were tested. The protocol used was the same as described in the next section below.

#### Optimized combination assay protocol

After incubation of the 384-well microplate, the contents of the wells were removed, and the wells were washed once with PBS. Then, 50 µL of the resazurin solution, diluted to 12 µg/mL immediately prior to use, was added to each well. Then the microplate was incubated at 37 °C for 150 min and fluorescence intensity was measured as relative fluorescence units (RFUs), using a Varioskan LUX plate reader (Thermo Fisher Scientific, Roskilde, Denmark), with the excitation wavelength λ_exc_ = 530 nm, and the emission wavelength λ_em_ = 590 nm.

Next, the crystal violet assay was initiated. First, the resazurin solution was removed from the wells and the microplate was allowed to dry for 30 min. Next, 50 µL of 0.023% crystal violet solution was added to the wells and stain was left in the wells for 30 min. The crystal violet was removed, and the wells were washed twice with sterile Milli-Q water. After washing, the microplate was left to dry for 45 min. Finally, 50 µL of 94 wt% ethanol was added to each well. The absorbance was measured at 570 nm with Multiskan GO (Thermo Fisher Scientific, Roskilde, Denmark).

#### Microplate assay uniformity and signal variability assessments

Once the combination assay was optimized, microplate assay uniformity and signal variability assessments, as described by Iversen et al. (2012), were performed using three types of samples yielding low (L, minimum), medium (M) and high (H, maximum) signals. Minimum signal wells contained only the medium, AU; medium signal wells contained bacteria at 10^6^ CFU/mL with 32 µg/mL tetracycline (TET); maximum signal wells contained only bacteria at 10^6^ CFU/mL. The interleaved-signal format was chosen as the microplate layout, in which each column had either L, H, or M signal wells. These assays were run with three different layouts: H-M-L, L-H-M, M-L-H. Resazurin and crystal violet assays were performed according to the protocol described above.

#### Compound screening

Prior to the screening of the compounds, the effect of DMSO on the growth of *E. coli* UMN026 was studied in the presence of 0–10% DMSO, a typical concentration range for testing the DMSO compatibility (Iversen et al. 2012). The final concentration of DMSO used in the compound screening was 1% for all conditions and controls tested.

Compound stocks, including garlic extracts, were prepared as described in the Sect. Materials. Bacterial biofilm was prepared as described in the Sect. Bacterial strain and growth conditions. Compounds were added to the microplate prior to bacteria. Biomek i7 automated workstation (Beckman Coulter, Brea, CA, USA) was used for dispensing compounds into assay microplates. Test concentration ranges for compounds were selected based on literature (Table 1). The aim was to use sub-MIC concentrations to test how the combination assay would detect changes in the biofilm formation of *E. coli* UMN026. For each tested compound, four concentrations were used. These concentrations were prepared as serial twofold dilutions. Four replicates of each tested compound condition were used per microplate. Biofilm assessment was carried out by using the protocol described in the Sect. Optimized combination assay protocol.

**Table 1:** Selection of compounds for screening and their test concentrations

Compound	Reported antibacterial/antibiofilm effects	Concentration range used in this study (µg/mL)	Reference
(+/-) - catechin	The biofilm formation of all the tested *E. coli* isolates was distorted at MIC (0.5–2 mg/mL).	125–1000	Jubair et al. (2022)
chrysin	*P. aeruginosa* PA01 biofilm formation was inhibited by 42.4% at 40 µg/mL.	5–40	Chang et al. (2021)
coumarin	*Porphyromonas gingivalis* biofilm was dispersed by c. 40.4% at 200 µM [c. 30 µg/mL].	7.5–60	He et al. (2022)
dispersin B	*S. epidermidis* biofilm was detached at 20 µg/mL.	1.8–14.4	Izano et al. (2008)
gallic acid	Biofilm formation of *E. coli* ATCC 25922 was inhibited by 70.83% at 1 mg/mL.	125–1000	Kang et al. (2018)
garlic extract	Biofilm of *E. coli* MG1655 was reduced by 50% at 0.1 mg/mL ethanolic extract.	37.5–300	Haindongo et al. (2021)
naringin	Biofilm of *Pseudomonas* CAP9 isolate was inhibited by 48.06% at 256 µg/mL.	64–512	Husain et al. (2021)
pyrogallol	Potential biofilm inhibition of 62% against *A. baumannii* was observed at 20 µg/mL.	2.5–20	Abirami et al. (2021)
quercetin	Biofilm formation of *E. coli* was inhibited by slightly over 50% at 6.25 µg/mL.	1.6–12.5	Vikram et al. (2010)
sodium acetate	Around 50% reduction in growth rate in *E. coli* was produced at 8 mM [656 µg/mL ]. No information on effects on biofilm.	70.25–562	Roe et al. (2002)
tannic acid	Biofilm of *S. aureus* ATCC 6538 was reduced by over 50% at 20 µg/mL.	2.5–20	Lee et al. (2013)
tannin(s)	The antibiofilm property of *Cinchona* extract, containing 2.2% of tannins, against *E. coli* PBio 730 was determined as minimal activity concentration 200 µg/mL.	25–200	Neumann et al. (2022)
trypsin	When 3-minute incubation time was used, 500 µg/mL was the minimum effective concentration for bovine trypsin to reduce extracellular polymeric substances (EPS) in a multispecies biofilm by over 50%.	6.25–50	Zhou et al. (2022)

## Data analysis

For the optimization of the assay, quality parameters Z′ factor (Z′), coefficient of variation (CV), signal-to-noise (S/N), and signal-to-background (S/B) were calculated using the formulae below (Inglese et al. 2007; Zhang et al. 1999):

1 $$Z^{\prime}=1-\frac{3 \sigma_{\max }+3 \sigma_{\min }}{\left|\mu_{\max }-\mu_{\min }\right|}$$

2 $$C V=\frac{\sigma}{\mu} \times 100 $$

3 $$S / N=\frac{\mu_{\max }-\mu_{\min }}{\sigma_{\min }}$$

4 $$S / B=\frac{\mu_{\max }}{\mu_{\min }}$$

Generally, an HTS assay is considered good when the Z′ ≥ 0.5 (Inglese et al. 2007; Zhang et al. 1999). It is well known that the crystal violet assay presents intrinsically larger variations between replicates (Amador et al. 2021) and slightly lower Z′ is acceptable. For the optimization and validation of the HTS assay, we used Z′ ≥ 0.4 as the reference, as described by Iversen et al. (2012).

In the assay validation phase, the equations below for Z′ and CV, which take into consideration the number of samples (*n*), were used as described by Iversen et al. (2012).

5 $$Z^{\prime}=\frac{\left(A V G_{M A X}-3 S D_{M A X} / \sqrt{n}\right)-\left(A V G_{M I N}+3 S D_{M I N} / \sqrt{n}\right)}{A V G_{M A X}-A V G_{M I N}}$$

6 $$C V=\frac{(S D / \sqrt{n})}{A V G} * 100$$

Standard deviation (SD) of the normalized medium signal (SD_MID_) was calculated as SD of all normalized medium signal values in a microplate after the raw signals were processed with Eq. 7. Drift effect was evaluated by dividing the difference of the average of two columns with the overall average of that signal type in the microplate, as described by Iversen et al. (2012).

Regarding compound screening assays, the results from each microplate were processed by normalizing the measured signal against the maximum signal, which is the untreated control. Compounds containing DMSO were normalized against DMSO-containing controls, while DMSO-free samples were normalized against DMSO-free controls. The results were first calculated as the percentage of signal against the untreated control using the equation below (Eq. 7) for each tested compound concentration. Next, inhibition percentages were calculated (Eq. 8). Average values were calculated from normalized results of individual wells.

7 $$\% \text { Activity }=\frac{\text { signal }_{\text {compound }}-A V G_{M I N}}{A V G_{M A X}-A V G_{M I N}} * 100 /$$

8 $$\% \text { Inhibition }=100-\% \text { Activity }$$

## Results and discussion

### Preliminary assay optimizations

Preliminary optimizations (see supplementary information) were done in clear 384-well microplates, with the resazurin and crystal violet assays carried out separately. One of the advantages of clear microplates is that the biofilm can be visually inspected by eye during the assay, and it is easier to adjust pipetting techniques. It is known that the choice of culture medium can have a direct impact on the type of biofilm formed by the bacteria (Paytubi et al. 2017). UPEC acquires nutrients from urine (Reitzer and Zimmern 2019), when it grows rapidly during an active infection (Forsyth et al. 2018). Therefore, AU was used as the culture medium for UPEC UMN026 biofilm studies throughout the assays.

Due to the small well size of the 384-well microplate, it is relevant to test and ensure that the working volume and the bacterial concentrations used are suitable. For example, if the working volume is too low, it may cause excessive evaporation, which must be prevented, as the *E. coli* UMN026 mature biofilm formation requires a 24-hour incubation. While testing the working volume, four bacterial concentrations (2.5 × 10^4^ – 1.0 × 10^6^ CFU/mL) were also tested to select a bacterial concentration which allowed for biofilm formation.

As a typical assay quality parameter employed in HTS, Z′ was used as the main guideline during the assay optimization process. For validating screening assays, Z′ is considered to be more reliable than the signal window (SW) (Iversen et al. 2006). Other parameters, such as CV, S/B, S/N, were also calculated and used as parameters on assessing optimal assay conditions. In general, for the validation of an HTS assay, the criterion is Z′ ≥ 0.4 (Iversen et al. 2012).

The optimal working volume for the resazurin assay was 50 µL, with the average Z′ at its highest, 0.60 ± 0.10, with the bacterial inoculum of 1 × 10^6^ CFU/mL (Fig. S1a). For the crystal violet assay, 50 µL was also the best working volume, although at the bacterial concentration of 1 × 10^6^ CFU/mL, the value of the Z′ was 0.17 ± 0.22 (Fig. S1b). Nonetheless, this concentration was chosen for further optimization steps, as it worked the best for the resazurin assay. The reason for this prioritization is discussed in the next section.

### Optimization of the combination assay

The combination assay was performed in the same microplate by starting with the resazurin assay followed by the crystal violet assay. One purpose was to reevaluate the conditions chosen based on the individually optimized assays described above. Three different concentrations of both resazurin and crystal violet were tested for each assay, in six combinations. Initial resazurin and crystal violet concentrations were selected based on an earlier work by Gilbert-Girard et al. (2020).

**Fig. 1:**
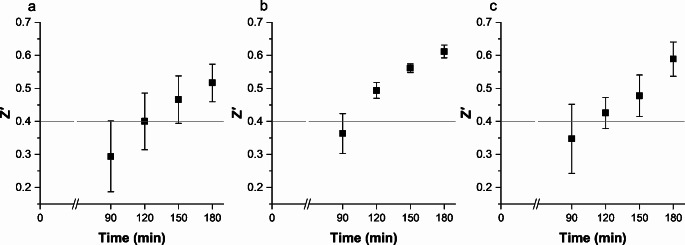
Effect of the resazurin concentration and incubation time on the Z′. Resazurin concentrations: **a**) 4 µg/mL, **b**) 12 µg/mL, **c**) 24 µg/mL. Results shown as average ± SD from three independent assays. 80 replicates per condition, and 22–24 for the control (AU only)

With all tested resazurin concentrations (4 µg/mL, 12 µg/mL, 24 µg/mL), it was observed that the Z′ improved as the incubation time increased. Resazurin at the concentration of 12 µg/mL resulted in the highest Z′ at 180 min (0.61 ± 0.02, Fig. 1b). With this concentration, there was also less variation between the experiments. The Z′ also reached acceptable level after 150 min incubation with the same resazurin concentration (0.56 ± 0.01, Fig. 1b). Thus, 12 µg/mL of resazurin with a 150-min incubation at 37 °C was selected, because 180-min was considered too long, and changing the plate type caused a drop in Z′ with 4 µg/mL, compared to preliminary data. Hence, the first condition above 0.4 was not selected in case further changes would cause additional drop.

Other parameters evaluated included CV for minimum and maximum signals, S/N, and S/B. Both S/N and S/B increased in positive correlation with the length of incubation time and were within acceptable values after two hours (Table S1). Higher S/N and S/B indicate that signal separates sufficiently from the background, and S/B over 2 is considered acceptable (Inglese et al. 2007). Maximum signal had larger CV than minimum signal, but all CV values remained below 20%, which is considered acceptable (Iversen et al. 2012).

**Fig. 2:**
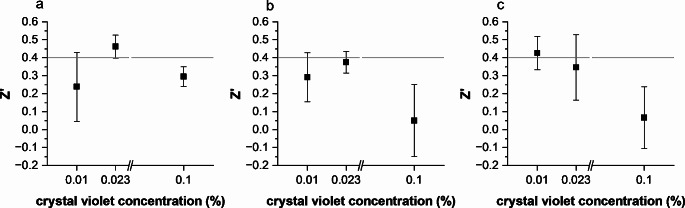
Effects of the crystal violet concentration on the Z′. After **a**) 4 µg/mL, **b**) 12 µg/mL, **c**) 24 µg/mL of resazurin. Results shown as average ± SD from three independent assays. 24 replicates per condition, and 6–8 for the control (AU only)

The crystal violet concentrations were tested against the three resazurin concentrations (Fig. 2). The highest Z′ (0.37 ± 0.06) was reached at the crystal violet concentration of 0.023%, when the assay was performed after 12 µg/mL resazurin. In most cases, we observed that when the Z′ was good, the other parameters were also at acceptable levels, in alignment with the guidelines (Table S2) (Iversen et al. 2012).

In the selection of optimal conditions for the combination assay, the resazurin assay was prioritized over the crystal violet assay, because the former performs with higher robustness and sensitivity than the latter, which intrinsically contains more variability (Amador et al. 2021). Previous research shows that resazurin staining had insignificant impact on the successive crystal violet assay when both assays were performed in the same microplate (Paytubi et al. 2017; Skogman et al. 2012).

A summary of the optimized parameters is displayed in Table 2. These were used during interleaved-signal format and compound screening assays.

**Table 2:** Optimized parameters of the combination assay for *E. coli* UMN026 biofilm assessment

	Parameter	Value
Bacterial inoculum	Bacterial concentration	1 × 10^6^ CFU/mL
	Total volume	50 µL/well
Resazurin assay	Concentration of resazurin	12 µg/mL
	Incubation time	150 min
Crystal violet assay	Concentration of crystal violet	0.023%
Both assays	Microplate type	sterile, untreated, black microplate with a transparent flat bottom

### Validation of the combination assay

Validation of the combination assay was carried out by performing interleaved-signal format assays to study the microplate uniformity, with the conditions described in Table 2.

**Fig. 3:**
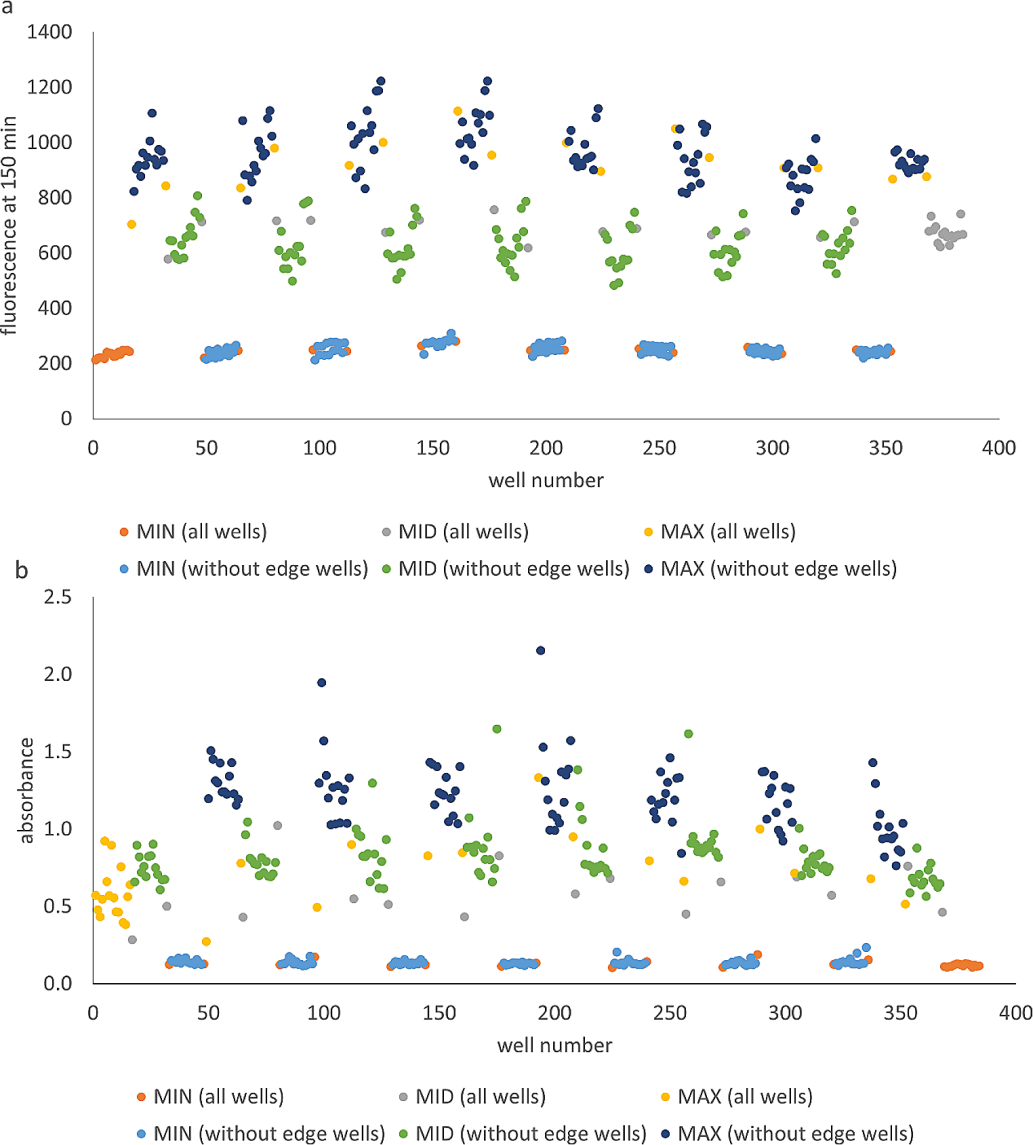
Validation of **(a)** resazurin and **(b)** crystal violet assays using signal variability assessment in interleaved 384-well microplate layout. Individual well numbers are arranged by column then row. The microplate contained three types of signals: minimum (0%, L), medium (50%, M), and maximum (100%, H). The number of replicates for each signal type is 128. The data shown here is from two representative microplates: **(a)** a resazurin assay microplate with L-H-M layout, and **(b)** a crystal violet assay microplate with H-M-L layout

**Table 3:** Assessment of uniformity and signal separation in the optimized combination screening assay^a^ performed in interleaved-signal format

**Resazurin assay**								
Layout	Z′	CV of MIN (%)	CV of MID (%)	CV of MAX (%)	S/N	S/B	MID signal compared to MAX (% of MAX)	Normalized SD of MID signal
L-H-M	0.5	7.7	11.9	9.9	36.9	3.8	64.7	10.4
H-M-L	0.4	3.6	13.5	16.9	166.2	7.0	60.3	9.5
M-L-H	0.6	5.2	7.0	6.9	39.8	3.1	76.8	8.0
**Crystal violet assay**								
Layout	Z′	CV of MIN (%)	CV of MID (%)	CV of MAX (%)	S/N	S/B	MID signal compared to MAX (% of MAX)	Normalized SD of MID signal
L-H-M	0.6	12.4	9.3	7.8	22.4	4.9	70.8	11.7
H-M-L	0.5	9.8	14.9	12.5	56.4	8.8	67.5	16.1
M-L-H	0.5	9.9	13.9	13	31.6	5.4	84.1	20.3

^a^The microplate uniformity was performed at maximum (H), medium (M) and minimum (L) signals, i.e., 0%, 50% and 100% of biofilm formation. Three microplates were assessed on three different days (*n* = 3)

For minimum (L), medium (M), and maximum (H) signals in both assays (Table 3), the CV remained below 20%, and the values of the Z′ remained ≥ 0.4 for all three tested assay layouts. In addition, the normalized SD_MID_ remained below or equal to 20. Furthermore, there was no fold shift of the normalized average MID signal, which is considered as another important criterion (Iversen et al. 2012).

Drift or edge effects below 20% and effects not occurring in a predominant pattern are considered insignificant (Iversen et al. 2012). In this study, drift tended to appear in top and bottom rows, while for outmost columns, the drift was at times negligible (Fig. 3a) and at times significant (Fig. 3b). However, it was observed visually that the evaporation of AU from the edge wells was significantly higher than from inner wells. Therefore, the edge wells were excluded from the results in the compound screening.

Based on the guidelines described by Iversen et al. (2012), the equations used for the Z′ and CV, during the validation of the combination assay, include *n*, as explained in the data analysis. According to validation data, the resazurin assay yielded reproducible results using *n* = 1, while for the crystal violet *n* = 2 is required (Table 3). This means that the final optimized combination assay requires a minimum of two replicate wells per sample.

### Effect of DMSO on the combination assay performance

DMSO, a common solvent used in compound libraries for HTS, may have an effect on bacterial growth, thus DMSO tolerance was assessed during the assay development. Summer et al. (2022) showed that DMSO (between 0.03 and 25%) can significantly inhibit the biofilm formation of *P. aeruginosa*. For UPEC UTI89, which is a well-studied biofilm-forming bacterial strain, a MIC of 16% for DMSO was found (Lim et al. 2012). As the response to DMSO varies even among the UPEC strains, DMSO dose-response effect on the biofilm formation of UPEC UMN026 was assessed.

At 0.1%, 0.5% and 1.0% DMSO, the signals (percentage of maximum) were 70.6 ± 3.4%, 66.8 ± 6.7%, and 68.2 ± 8.7%, respectively in the resazurin assay, and 64.2 ± 11.8%, 62.9 ± 10.2%, and 65.0 ± 12.8%, respectively in the crystal violet assay. The final DMSO concentration of 1% was chosen, as the effect on the signal was not significantly different from lower concentrations, when compared to DMSO-free control (Fig. S2).

### Compound screening

The optimized 384-well-based combination assay platform was employed to screen a set of 14 compounds and a garlic extract sample (Table 1) against *E. coli* UMN026 biofilm. The selection of compounds was based on (a) known inhibitory activity of compounds against bacterial biofilm, as per literature findings, and (b) availability of compounds from our *in-house* collection. This screening aimed at evaluating the efficacy of compounds to prevent biofilm formation when incubated for 24 h. Each compound was tested in four concentrations, and subsequent biofilm inhibitory effects were analyzed in terms of biomass and metabolic activity of biofilm cells.

**Fig. 4:**
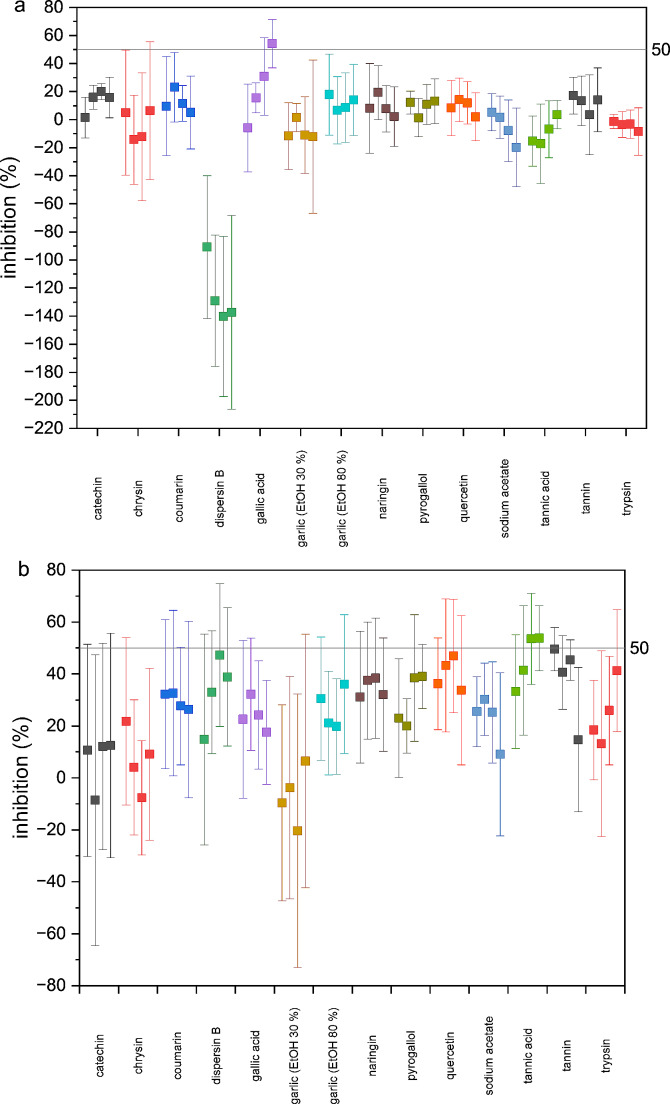
Compound screening results from the optimized UPEC UMN026 biofilm assay. **a**) Metabolic activity (fluorescence) **b**) Biomass (absorbance). Data points are averages ± SD from three independent assays, with four replicates for compounds, and 12–16 replicates for the controls. Final DMSO concentration was 1%. Results were normalized to the untreated control (maximal bacterial growth) and expressed as inhibition percentages. Each compound was tested at four different concentrations. Each leftmost data point is the lowest concentration for each compound sample

To help with the interpretation of screening results we established a threshold for antibiofilm activity, i.e. compounds demonstrating a minimum inhibition of 50% in either biofilm parameter measured, were classified as active. Most of the tested samples were natural products (Table 1) and overall had no significant antibiofilm activity at the tested concentrations (Fig. 4, Table S3). Moreover, significant effects were mostly observed in terms of biomass reduction, and not in metabolic activity of cells. It is noteworthy that *E. coli* UMN026 is an MDR, strong biofilm former and highly virulent strain (Kot 2019; Zhao et al. 2020), consequently a hard-to-defeat target. Still, natural products remain central to drug discovery (Dzobo 2022), and in the case of UPEC, medicinal plants have been studied for their antibiofilm activities (Bhandari et al. 2021; Vysakh et al. 2020). For example, medicinal plants found in Nepal, namely *Calotropis gigantea* (L.) Dryand., *Eclipta prostrata* L., *Moringa oleifera* Lam., were found to inhibit biofilm by more than 60% in most of the tested clinical UPEC strains which form strong biofilm (Bhandari et al. 2021). To the best of our knowledge, the selected compounds have not been previously assessed against *E. coli* UMN026 biofilm.

Eight compounds used in the screening assays were polyphenols. Most active groups were flavonoids followed by the phenolic acids. During the screening assays, precipitation of chrysin was observed, thus the screening results of this compound are not discussed here.

Our results (Fig. 4) showed that quercetin (6.25 µg/mL), tannic acid (10 µg/mL) and tannin (25 µg/mL) were the most potent ones, showing significant inhibitory activity against *E. coli* UMN026 biofilm, with a decrease in biomass by 46.9 ± 21.9%, 53.5 ± 17.5%, and 49.6 ± 8.3%, respectively. Inhibitions did not improve with increasing concentrations of the compounds (i.e., up to 8 × higher). A study by Vikram et al. (2010) showed that quercetin at 6.25 µg/mL inhibited the biofilm formation of *E. coli* O157:H7 by slightly over 50%. The effects of tannic acid were in line with previous findings by Lee et al. (2013), who reported that tannic acid at 20 µg/mL reduced biofilm of *S. aureus* ATCC 6538 by over 50%. A previous study with *Cinchona* bark extract at 200 µg/mL (with a tannin content of 2.2%) also resulted in weak activity against *E. coli* PBio 730 biofilm (Neumann et al. 2022). Our results showed higher activity, most likely because we used pure tannin.

In our study naringin showed a slightly weaker effect and high variability (i.e., at 256 µg/mL a 38.3 ± 23.2% decrease in biomass). One earlier study showed that at the same concentration the biofilm of *Pseudomonas* CAP9 was reduced by 48.06% (Husain et al. 2021). Finally, catechin did not show any significant antibiofilm activity against *E. coli* UMN026, contrary to previously reported data on clinical UTI *E. coli* isolates, in which an average of 82% biofilm inhibition for the isolates was observed when tested at MIC (1 mg/mL for 15 isolates, 0.5 mg/mL for two isolates, and 2 mg/mL for one isolate) (Jubair et al. 2022).

Pyrogallol (20 µg/mL) was also active, and decreased biomass of *E. coli* UMN026 by 39.0 ± 12.4% (Fig. 4, Table S3). Abirami et al. (2021) reported that pyrogallol at 20 µg/mL had a potential biofilm inhibition of 62% against *A. baumannii*, a gram-negative bacterium. One earlier study reported that gallic acid at 1 mg/mL showed a great impact on biofilm formed by *E. coli* ATCC 25922, with a decrease in biomass by 70.83% (Kang et al. 2018), but in our study its inhibitory effect was < 20%. Peculiarly, a significant decrease in the metabolic activity of UMN026 biofilm cells was seen (54.1 ± 17.2%, Fig. 4, Table S3). This is an important outcome as biomass does not take into account living and viable cells. Kang et al. (2018) used a different *E. coli* strain, which is a reference CLSI strain and does not harbor any antibiotic resistance gene.

Based on current findings, we cannot infer that either coumarin or garlic extract possess antibiofilm property. Albeit biomass reduction was observed, variation between replicate assays does not support a conclusion (i.e., biomass reduction of coumarin at 60 µg/mL was 26.3 ± 33.9% and garlic extract 80% EtOH at 300 µg/mL was 36.1 ± 26.8%). He et al. (2022) demonstrated that coumarin dispersed c. 40.4% of *Porphyromonas gingivalis* biofilm at 200 µM [c. 30 µg/mL]. Garlic is long known in traditional medicine for its antibacterial effect, attributed mostly to allicin (Ankri and Mirelman 1999). Its antibiofilm role has been recently shown against *E. coli* MG1655, i.e., 100 µg/mL caused 50% reduction in biomass (Haindongo et al. 2021).

In our study dispersin B significantly increased the metabolic activity of *E. coli* UMN026 biofilm cells while reducing the biomass (Fig. 4). The latter effect was anticipated as dispersin B is an enzyme that causes dispersal of the biofilm by degrading surface polysaccharides of the biofilm matrix, which has been demonstrated in *Staphylococcus epidermidis* strains (Izano et al. 2008). The stimulating effect of dispersin B, at all tested concentrations, by over 90% on the metabolic activity of biofilm cells was not expected. In contrast to the planktonic cells, it is a characteristic of the bacterial cells in the biofilm to grow slowly (Brown et al. 1988). Therefore, dispersin B probably exposed dormant cells, by dispersing the upper biofilm layer, causing a change in the cellular metabolic pattern. Hence, while our initial threshold is at 50% inhibition for both measurements, stimulating effects on the metabolic activity may prove to be worth studying further, especially if there is a concurrent decrease in the biomass.

Sodium acetate was the only compound whose antibiofilm property has not been yet described in the literature. Still, sodium acetate is known for inhibiting *E. coli* growth by causing homocysteine accumulation within the cells (Roe et al. 2002). According to Roe et al. (2002), sodium acetate at 8 mM [656 µg/mL] decreased 50% of planktonic *E. coli* growth rate. Our results did not show any antibiofilm activity up to 562 µg/mL (Fig. 4, Table S3). Bovine trypsin at 500 µg/mL, with a 3-minute incubation, has shown to decrease the biomass (EPS) of multispecies biofilm (i.e., *Actinomyces viscosus, Fusobacterium nucleatum* subsp. *polymorpha, Aggregatibacter actinomycetemcomitans*, and *P. gingivalis*) by over 50% (Zhou et al. 2022). We could also observe the effect of porcine trypsin (50 µg/mL) on *E. coli* UMN026 biomass with a reduction of 41.3 ± 23.4%.

#### Performance of the combination assay in compound screening

Over the years several methods for studying biofilms have emerged (Peeters et al. 2008). Amongst these, static biofilm using 96-well microplate platform and quantification assays based on colorimetric, i.e., crystal violet, and fluorescence, i.e., resazurin measurements, are still considered the methods of choice in screening campaigns for e.g., biofilm inhibitors. There is a need for improved HTS assays that would overcome the use of a two-plate system while increasing the screening capacity and saving compounds and time.

In this study we optimized and validated an antibiofilm screening platform by integrating resazurin and crystal violet assay in a 384-well microplate format. Clinical UPEC UMN026, a MDR strain, was used as a biofilm-forming model. The method was optimized for initial bacterial concentrations, working volumes, resazurin and crystal violet concentrations, and incubation times.

A few limitations observed were related to sensitivity to initial bacterial concentrations, which critically affected the initial values of untreated controls, being evident in plate-to-plate variations present in Table S4. Specifically, in the second assay, there were many compounds leading to over 50% reduction in biomass. This result should be evaluated with caution, because in this assay the maximum signal of the untreated control was lower, potentially due to lower bacterial amounts in the inoculum, thus potentially resulting in less biofilm formed. This could have perhaps affected the outcome caused by some of the compounds.

Our 384-well microplate combination assay proved to be a good tool, showing high sensitivity, high S/B, and was overall statistically robust. In general, CV remained below 20% for resazurin assays but was occasionally higher for crystal violet assays (Table S4). Crystal violet-based assay is notoriously known for its high variability (Amador et al. 2021). The medium signal-producing control, tetracycline at 32 µg/mL, was included as positive control in all the compound screening microplates, with variation in it observed across microplates (Table S5). This is not unexpected, since the signal of the untreated control had variation as well (Table S6). We demonstrated that our platform can be successfully applied for measuring changes in biofilm of UPEC UMN026 grown under an UTI-scenario (i.e., AU as assay medium).

## Conclusions

The optimized and validated HTS method for a 384-well microplate using a combination assay, performed with resazurin and crystal violet, established in this study, is suitable for HTS for antibiofilm agents against uropathogenic *E. coli* UMN026. To mimic in vivo environment that UPEC inhabits, artificial urine was used as the culture medium. The resazurin assay proved to have high robustness, both in terms of repeatability of the results and minimal variation in replicate samples. Crystal violet staining could detect biomass decreases, despite having more often plate-specific variation than the resazurin assay. The combination assay in its current form requires plenty of manual handling, which could limit the screening of very large libraries of compounds, as only one microplate can be processed at a time. In future, this could be improved with laboratory automation.

Since the growth of bacterial cells in the biofilm is naturally slow (Brown et al. 1988), potential hits might present themselves as a combination of increase in the metabolic activity with the concurrent decrease in the biomass, as it was in the case of dispersin B. Therefore, one should not only look for effects that cause a decrease in signal when screening is performed.

Overall, the HTS combination assay performed well in terms of usability, considering the fact that UPEC biofilm in its pellicle form, is difficult to study, as the biofilm can break up easily. It detected compounds displaying antibiofilm activity against UPEC UMN026 strain at sub-inhibitory concentrations. Thus, this HTS combination assay is suitable for initial screening of compounds of interest as it provides more information than either of the assays alone, and the readouts can be doubled, which increases timewise efficiency of a screening. As variation was observed between repeat assays of compound screening, confirmation of potential effects with 96-well microplate assay should be considered.

### Electronic supplementary material

Below is the link to the electronic supplementary material.

Supplementary Material 1

## Data Availability

The datasets generated during and/or analyzed during the current study are available from the corresponding author on reasonable request.
