# Supplementary material for: High-throughput combination assay for studying biofilm formation of uropathogenic Escherichia coli
Source: Arch Microbiol. 2024 Jul 5;206(8):344. doi: 10.1007/s00203-024-04029-w (PMC11226472; doi:10.1007/s00203-024-04029-w)
Supplement: Supplementary file 1 — Supplementary Material 1 [file 203_2024_4029_MOESM1_ESM.docx]

# Supplementary information

HIGH-THROUGHPUT COMBINATION ASSAY FOR STUDYING BIOFILM FORMATION OF UROPATHOGENIC *ESCHERICHIA COLI*

Archives of Microbiology

M. Li,^a^ C. D. Cruz,^a^ P. Ilina,^a^ and P. Tammela^a,*^

*^a^ Drug Research Program, Division of Pharmaceutical Biosciences, Faculty of Pharmacy, University of Helsinki, Finland*

*Corresponding author, email address: paivi.tammela@helsinki.fi

## Assay for optimization of working volume and the bacterial inoculum

Bacterial concentrations tested were 1 × 10^6^ CFU/mL, 2.5 × 10^5^ CFU/mL, 7 × 10^4^ CFU/mL, and 2.5 × 10^4^ CFU/mL at two different working volumes: 40 µL and 50 µL. Working volume refers to the total volume in a well after the addition of the medium and bacteria, prior to the 24-hour incubation. Clear 384-well microplates (Nunc™ 242757, Thermo Fisher Scientific, Roskilde, Denmark) were used.

Resazurin assay was performed with 4 µg/mL resazurin with 120 min incubation. Crystal violet assay was performed with 0.01 % crystal violet with 30 min staining time. These conditions have been previously studied and used in our research group (Cruz et al. 2018).

**Fig. S1** Effect of bacterial concentration and working volume on Z′ **a**) Resazurin assay **b**) Crystal violet assay. For the resazurin assay, the measurements were taken at 120 min of incubation after adding the resazurin dye. Data points are averages ± SD from two independent assays, number of technical replicates for test wells and background control wells were 30 and 34, respectively, in both resazurin and crystal violet assays

**Fig. S2** Effect of DMSO on *E. coli* UMN026 biofilm. The signals (fluorescence and absorbance) were normalized to untreated control (maximal growth). Data from one assay with 28 technical replicates per condition

**Table S1** Effects of the resazurin concentration and incubation time on the coefficient of variation (CV), signal-to-noise (S/N) and signal-to background (S/B) parameters. Same conditions as in Fig. 1

| Resazurin (µg/mL) | Time (min) | CV of MIN (%) | CV of MAX (%) | S/N | S/B |
| --- | --- | --- | --- | --- | --- |
| 4 | 90 | 3.3 ± 0.5 | 8.7 ± 1.8 | 25.1 ± 6.6 | 1.8 ± 0.1 |
|  | 120 | 3.3 ± 0.4 | 8.7 ± 1.6 | 32.6 ± 7.3 | 2.1 ± 0.1 |
|  | 150 | 3.4 ± 0.3 | 8.7 ± 1.5 | 39.6 ± 8.5 | 2.3 ± 0.2 |
|  | 180 | 3.5 ± 0.8 | 8.4 ± 1.2 | 46.3 ± 14.9 | 2.5 ± 0.2 |
| 12 | 90 | 1.9 ± 0.8 | 9.4 ± 2.6 | 59.2 ± 29.4 | 2.0 ± 0.4 |
|  | 120 | 1.9 ± 0.9 | 9.1 ± 1.9 | 90.0 ± 41.8 | 2.5 ± 0.7 |
|  | 150 | 1.8 ± 0.9 | 8.6 ± 1.0 | 112.0 ± 47.9 | 2.8 ± 0.8 |
|  | 180 | 1.8 ± 0.9 | 8.1 ± 0.7 | 136.9 ± 68.6 | 3.1 ± 0.8 |
| 24 | 90 | 1.7 ± 0.2 | 12.2 ± 3.4 | 85.4 ± 26.3 | 2.4 ± 0.3 |
|  | 120 | 1.7 ± 0.3 | 12.7 ± 2.5 | 135.5 ± 55.3 | 3.3 ± 0.7 |
|  | 150 | 1.8 ± 0.3 | 12.8 ± 3.1 | 194.5 ± 107.4 | 4.3 ± 1.4 |
|  | 180 | 1.9 ± 0.3 | 10.3 ± 1.5 | 229.9 ± 141.3 | 5.1 ± 2.1 |

**Table S2** Effects of the crystal violet concentration on CV, S/N, and S/B parameters. Same conditions as in Fig. 2

| Resazurin (µg/mL) | Crystal violet (%) | CV of MIN (%) | CV of MAX (%) | S/N | S/B |
| --- | --- | --- | --- | --- | --- |
| 4 | 0.010 | 23.4 ± 11.9 | 15.4 ± 2.2 | 22.6 ± 17.0 | 5.0 ± 0.4 |
|  | 0.023 | 17.7 ± 6.5 | 11.0 ± 1.3 | 25.0 ± 7.1 | 5.1 ± 0.5 |
|  | 0.100 | 20.7 ± 2.9 | 12.3 ± 1.6 | 14.3 ± 1.5 | 3.9 ± 0.3 |
| 12 | 0.010 | 14.4 ± 2.5 | 16.6 ± 4.1 | 31.1 ± 5.3 | 5.4 ± 0.3 |
|  | 0.023 | 16.3 ± 4.8 | 12.2 ± 3.5 | 23.8 ± 13.4 | 4.5 ± 0.8 |
|  | 0.100 | 30.8 ± 9.0 | 12.2 ± 2.2 | 7.8 ± 2.8 | 3.3 ± 0.5 |
| 24 | 0.010 | 12.1 ± 4.8 | 14.4 ± 3.3 | 59.2 ± 43.6 | 6.8 ± 1.3 |
|  | 0.023 | 17.6 ± 1.6 | 15.8 ± 5.1 | 31.4 ± 2.3 | 6.5 ± 0.1 |
|  | 0.100 | 29.9 ± 5.6 | 16.2 ± 1.9 | 11.2 ± 4.2 | 4.2 ± 0.5 |

**Table S3** Effect of compounds^a^ expressed as inhibition (%), in comparison to the maximum signal of the untreated control

| Compound | Concentration (µg/mL) | Fluorescence | Absorbance |
| --- | --- | --- | --- |
| catechin | 125 | 1.5 ± 14.5 | 10.6 ± 40.9 |
|  | 250 | 16.0 ± 8.6 | -8.6 ± 55.9 |
|  | 500 | 20.1 ± 5.6 | 12.1 ± 39.7 |
|  | 1000 | 15.8 ± 14.5 | 12.4 ± 43.2 |
| chrysin | 5 | 4.9 ± 44.6 | 21.8 ± 32.2 |
|  | 10 | -14.3 ± 31.7 | 4.0 ± 26.0 |
|  | 20 | -12.1 ± 45.5 | -7.7 ± 22.0 |
|  | 40 | 6.4 ± 49.1 | 9.1 ± 33.1 |
| coumarin | 7.5 | 9.6 ± 35.3 | 32.2 ± 28.7 |
|  | 15 | 23.2 ± 24.8 | 32.6 ± 31.9 |
|  | 30 | 11.6 ± 12.8 | 27.7 ± 22.6 |
|  | 60 | 5.1 ± 25.9 | 26.3 ± 33.9 |
| dispersin B | 1.8 | **-90.8 ± 50.8** | 14.8 ± 40.6 |
|  | 3.6 | **-129.1 ± 46.7** | 33.0 ± 23.6 |
|  | 7.2 | **-140.2 ± 57.0** | 47.3 ± 27.4 |
|  | 14.4 | **-137.3 ± 69.0** | 38.9 ± 26.6 |
| gallic acid | 125 | -5.9 ± 31.2 | 22.5 ± 30.4 |
|  | 250 | 15.5 ± 10.7 | 32.2 ± 21.6 |
|  | 500 | 30.8 ± 27.7 | 24.2 ± 20.8 |
|  | 1000 | **54.1 ± 17.2** | 17.5 ± 20.0 |
| garlic extract (EtOH 30 %) | 37.5 | -11.7 ± 23.8 | -9.7 ± 37.7 |
|  | 75 | 1.5 ± 10.1 | -3.8 ± 42.8 |
|  | 150 | -11.0 ± 27.4 | -20.4 ± 52.7 |
|  | 300 | -12.1 ± 54.6 | 6.5 ± 48.8 |
| garlic extract (EtOH 80 %) | 37.5 | 17.9 ± 28.8 | 30.5 ± 23.7 |
|  | 75 | 6.7 ± 24.0 | 21.1 ± 20.0 |
|  | 150 | 8.6 ± 24.7 | 19.8 ± 18.3 |
|  | 300 | 14.0 ± 25.3 | 36.1 ± 26.8 |
| naringin | 64 | 8.1 ± 31.9 | 31.1 ± 25.4 |
|  | 128 | 19.3 ± 19.3 | 37.5 ± 22.5 |
|  | 256 | 7.7 ± 16.4 | 38.3 ± 23.2 |
|  | 512 | 2.2 ± 21.2 | 32.1 ± 21.9 |
| pyrogallol | 2.5 | 12.2 ± 8.2 | 23.0 ± 22.9 |
|  | 5 | 1.4 ± 13.7 | 20.0 ± 10.5 |
|  | 10 | 10.9 ± 14.2 | 38.4 ± 24.4 |
|  | 20 | 13.1 ± 15.9 | 39.0 ± 12.4 |
| quercetin | 1.5625 | 8.3 ± 19.6 | 36.2 ± 17.7 |
|  | 3.125 | 14.2 ± 15.5 | 43.3 ± 25.7 |
|  | 6.25 | 11.9 ± 15.0 | 46.9 ± 21.9 |
|  | 12.5 | 2.0 ± 17.0 | 33.8 ± 28.7 |
| sodium acetate | 70.25 | 5.3 ± 13.2 | 25.5 ± 13.5 |
|  | 140.5 | 1.7 ± 15.1 | 30.3 ± 13.9 |
|  | 281 | -7.8 ± 21.9 | 25.2 ± 19.4 |
|  | 562 | -19.7 ± 27.9 | 9.0 ± 31.4 |
| tannic acid | 2.5 | -15.2 ± 17.9 | 33.2 ± 22.0 |
|  | 5 | -17.1 ± 28.3 | 41.4 ± 24.9 |
|  | 10 | -6.8 ± 20.3 | **53.5 ± 17.5** |
|  | 20 | 3.5 ± 10.2 | **53.8 ± 12.5** |
| tannin | 25 | 17.2 ± 13.1 | 49.6 ± 8.3 |
|  | 50 | 13.4 ± 17.5 | 40.6 ± 14.2 |
|  | 100 | 3.5 ± 28.5 | 45.4 ± 7.8 |
|  | 200 | 14.1 ± 22.7 | 14.7 ± 27.8 |
| trypsin | 6.25 | -1.4 ± 5.0 | 18.4 ± 19.2 |
|  | 12.5 | -3.4 ± 9.2 | 13.2 ± 35.8 |
|  | 25 | -3.1 ± 10.1 | 25.9 ± 20.9 |
|  | 50 | -8.4 ± 16.9 | 41.3 ± 23.4 |

^a^The values are averages ± SD from three independent assays. Four replicates per condition. Each compound was tested at four concentrations. Over 50 % effects are bolded

**Table S4** Signal data from compound screening^a^

|  |  | Fluorescence | | | | | | | | | Absorbance | | | | | | | | | |
| --- | --- | --- | --- | --- | --- | --- | --- | --- | --- | --- | --- | --- | --- | --- | --- | --- | --- | --- | --- | --- |
|  |  | Assay 1 | | | Assay 2 | | | Assay 3 | | | Assay 1 | | | Assay 2 | | | Assay 3 | | |  |
|  | Concentration (µg/mL) | % of max | CV (%) | % of max | | CV (%) | % of max | | CV (%) | % of max | | CV (%) | % of max | | CV (%) | % of max | | CV (%) |  |  |
| catechin | 125 | 105.1 ± 7.1 | 6.7 | 81.9 ± 11.5 | | 14.0 | 108.6 ± 5.2 | | 4.8 | 130.7 ± 43.2 | | **33.1** | 72.5 ± 24.9 | | **34.4** | 65.0 ± 13.5 | | **20.7** |  |  |
|  | 250 | 89.4 ± 4.4 | 5.0 | 75.1 ± 5.1 | | 6.8 | 87.5 ± 7.9 | | 9.0 | **182.0 ± 18.7** | | 10.3 | 64.8 ± 10.2 | | 15.8 | 79.0 ± 10.9 | | 13.9 |  |  |
|  | 500 | 84.6 ± 3.7 | 4.3 | 73.3 ± 1.0 | | 1.4 | 81.9 ± 2.9 | | 3.6 | 131.8 ± 40.1 | | **30.4** | 59.8 ± 12.2 | | **20.4** | 72.1 ± 8.3 | | 11.6 |  |  |
|  | 1000 | 80.0 ± 6.0 | 7.5 | 72.4 ± 9.0 | | 12.5 | 100.2 ± 9.9 | | 9.9 | 128.1 ± 49.9 | | **38.9** | 51.0 ± 12.3 | | **24.1** | 83.6 ± 14.5 | | 17.4 |  |  |
| chrysin | 5 | **46.7 ± 35.1** | **75.2** | 99.0 ± 5.4 | | 5.4 | 139.6 ± 15.6 | | 11.2 | **41.8 ± 7.0** | | 16.8 | 82.4 ± 18.1 | | **21.9** | 110.5 ± 15.8 | | 14.3 |  |  |
|  | 10 | 92.2 ± 39.5 | **42.8** | 105.4 ± 4.3 | | 4.1 | 145.3 ± 9.2 | | 6.3 | 86.4 ± 46.1 | | **53.4** | 103.6 ± 10.7 | | 10.3 | 97.8 ± 5.7 | | 5.8 |  |  |
|  | 20 | 75.9 ± 50.2 | **66.2** | 104.7 ± 6.5 | | 6.2 | **155.8 ± 25.4** | | 16.3 | 89.8 ± 16.1 | | 18.0 | 113.9 ± 23.6 | | **20.7** | 119.2 ± 17.6 | | 14.7 |  |  |
|  | 40 | **31.4 ± 10.8** | **34.3** | 107.9 ± 2.4 | | 2.2 | 141.4 ± 15.1 | | 10.7 | 61.8 ± 12.9 | | **20.8** | 113.5 ± 42.7 | | **37.6** | 97.4 ± 13.0 | | 13.3 |  |  |
| coumarin | 7.5 | 52.0 ± 7.7 | 14.7 | 88.9 ± 8.1 | | 9.1 | 130.4 ± 18.7 | | 14.4 | 77.4 ± 23.9 | | **30.8** | **37.4 ± 8.8** | | **23.5** | 88.4 ± 21.3 | | **24.1** |  |  |
|  | 15 | 51.4 ± 6.8 | 13.3 | 76.3 ± 1.4 | | 1.8 | 102.8 ± 20.9 | | **20.3** | 67.7 ± 19.1 | | **28.3** | **37.8 ± 12.3** | | **32.5** | 96.7 ± 30.1 | | **31.1** |  |  |
|  | 30 | 94.1 ± 4.8 | 5.1 | 77.7 ± 5.0 | | 6.4 | 93.4 ± 18.1 | | 19.4 | 80.8 ± 28.8 | | **35.7** | 50.5 ± 6.6 | | 13.0 | 85.7 ± 5.8 | | 6.8 |  |  |
|  | 60 | 92.0 ± 15.9 | 17.2 | 68.5 ± 5.8 | | 8.5 | 124.1 ± 10.1 | | 8.1 | 101.8 ± 13.5 | | 13.3 | **32.3 ± 17.3** | | **53.7** | 87.0 ± 12.6 | | 14.5 |  |  |
| dispersin B | 1.8 | **248.9 ± 12.7** | 5.1 | 134.1 ± 22.9 | | 17.1 | **189.4 ± 3.5** | | 1.8 | 84.8 ± 10.8 | | 12.8 | **41.2 ± 6.4** | | 15.5 | 129.7 ± 25.8 | | 19.9 |  |  |
|  | 3.6 | **258.2 ± 10.3** | 4.0 | **244.2 ± 61.5** | | **25.2** | **184.8 ± 8.9** | | 4.8 | 60.5 ± 21.5 | | **35.5** | 56.5 ± 26.3 | | **46.5** | 84.0 ± 17.4 | | **20.7** |  |  |
|  | 7.2 | **233.1 ± 12.9** | 5.5 | **308.9 ± 16.9** | | 5.5 | **178.8 ± 9.2** | | 5.1 | **39.6 ± 23.4** | | **59.1** | 75.2 ± 27.6 | | **36.7** | **43.4 ± 20.7** | | **47.7** |  |  |
|  | 14.4 | **232.3 ± 11.7** | 5.0 | **318.9 ± 12.8** | | 4.0 | **160.7 ± 20.3** | | 12.6 | 55.1 ± 10.0 | | 18.1 | 86.8 ± 28.9 | | **33.3** | **41.5 ± 14.9** | | **35.8** |  |  |
| gallic acid | 125 | 97.8 ± 3.6 | 3.7 | 78.4 ± 8.1 | | 10.3 | 141.5 ± 26.5 | | 18.7 | 98.9 ± 29.5 | | **29.8** | **42.8 ± 4.8** | | 11.2 | 90.7 ± 6.3 | | 6.9 |  |  |
|  | 250 | 84.9 ± 3.5 | 4.1 | 73.7 ± 2.2 | | 2.9 | 94.9 ± 10.1 | | 10.6 | 88.2 ± 15.7 | | 17.8 | **43.3 ± 6.4** | | 14.8 | 71.9 ± 7.0 | | 9.7 |  |  |
|  | 500 | 58.5 ± 5.4 | 9.2 | **44.2 ± 4.0** | | 9.1 | 105.0 ± 8.1 | | 7.7 | 85.3 ± 29.7 | | **34.8** | 60.7 ± 12.7 | | **20.9** | 81.4 ± 8.8 | | 10.8 |  |  |
|  | 1000 | 55.1 ± 6.5 | 11.7 | **24.1 ± 8.3** | | **34.5** | 58.4 ± 4.7 | | 8.0 | 101.7 ± 18.8 | | 18.5 | 63.5 ± 3.1 | | 4.9 | 82.2 ± 11.7 | | 14.2 |  |  |
| garlic extract (EtOH 30 %) | 37.5 | 90.2 ± 7.6 | 8.4 | 134.2 ± 25.7 | | 19.2 | 110.7 ± 8.0 | | 7.3 | 84.6 ± 50.9 | | **60.1** | 128.7 ± 32.4 | | **25.2** | 115.7 ± 14.7 | | 12.7 |  |  |
|  | 75 | 99.5 ± 4.5 | 4.5 | 94.4 ± 6.1 | | 6.5 | 101.6 ± 16.8 | | 16.6 | 77.9 ± 4.4 | | 5.7 | 93.9 ± 49.0 | | **52.2** | 139.6 ± 39.6 | | **28.4** |  |  |
|  | 150 | 122.2 ± 37.4 | **30.6** | 105.0 ± 28.6 | | **27.2** | 105.9 ± 16.9 | | 16.0 | 141.1 ± 66.0 | | **46.8** | 81.0 ± 41.5 | | **51.3** | 139.2 ± 31.7 | | **22.8** |  |  |
|  | 300 | 142.4 ± 83.8 | **58.9** | 89.2 ± 43.2 | | **48.4** | 104.8 ± 7.9 | | 7.5 | 137.2 ± 44.4 | | **32.4** | **39.8 ± 5.4** | | 13.5 | 103.6 ± 13.8 | | 13.3 |  |  |
| garlic extract (EtOH 80 %) | 37.5 | **49.5 ± 7.7** | 15.5 | 81.1 ± 4.8 | | 5.9 | 115.6 ± 6.2 | | 5.4 | 64.1 ± 7.8 | | 12.2 | 50.4 ± 15.2 | | **30.2** | 93.9 ± 21.4 | | **22.8** |  |  |
|  | 75 | 84.0 ± 30.0 | **35.7** | 81.5 ± 5.0 | | 6.2 | 114.4 ± 17.0 | | 14.8 | 88.4 ± 5.8 | | 6.6 | 55.5 ± 15.0 | | **27.0** | 92.8 ± 9.8 | | 10.6 |  |  |
|  | 150 | 77.1 ± 24.7 | **32.1** | 83.7 ± 6.6 | | 7.9 | 113.5 ± 24.0 | | **21.2** | 71.4 ± 16.3 | | **22.8** | 75.7 ± 22.2 | | **29.4** | 93.5 ± 10.2 | | 10.9 |  |  |
|  | 300 | 81.3 ± 33.7 | **41.4** | 73.8 ± 16.3 | | **22.1** | 102.8 ± 18.5 | | 18.0 | 65.7 ± 12.0 | | 18.3 | **34.5 ± 14.7** | | **42.7** | 91.6 ± 9.1 | | 9.9 |  |  |
| naringin | 64 | 69.6 ± 23.6 | **33.9** | 85.4 ± 7.0 | | 8.2 | 120.8 ± 36.1 | | **29.8** | 69.1 ± 15.8 | | **22.9** | **45.8 ± 11.2** | | **24.4** | 91.9 ± 23.8 | | **25.9** |  |  |
|  | 128 | 74.6 ± 26.1 | **35.0** | 74.3 ± 4.0 | | 5.4 | 93.1 ± 19.1 | | **20.5** | 76.2 ± 11.6 | | 15.2 | **33.6 ± 2.4** | | 7.3 | 77.8 ± 6.4 | | 8.2 |  |  |
|  | 256 | 96.5 ± 3.7 | 3.8 | 73.2 ± 2.2 | | 3.0 | 107.1 ± 13.1 | | 12.3 | 78.7 ± 15.9 | | **20.2** | **32.8 ± 2.0** | | 6.1 | 73.4 ± 5.3 | | 7.2 |  |  |
|  | 512 | 101.2 ± 4.0 | 3.9 | 77.5 ± 14.2 | | 18.3 | 114.8 ± 22.0 | | 19.2 | 86.3 ± 5.4 | | 6.3 | **40.6 ± 6.8** | | 16.8 | 76.9 ± 11.0 | | 14.3 |  |  |
| pyrogallol | 2.5 | 90.1 ± 3.4 | 3.8 | 80.0 ± 3.1 | | 3.9 | 93.2 ± 9.9 | | 10.6 | 82.8 ± 7.4 | | 9.0 | **49.5 ± 11.5** | | **23.3** | 98.8 ± 7.0 | | 7.1 |  |  |
|  | 5 | 101.4 ± 8.6 | 8.5 | 84.9 ± 7.1 | | 8.4 | 109.5 ± 12.2 | | 11.2 | 83.5 ± 7.6 | | 9.1 | 74.3 ± 16.4 | | **22.0** | 82.2 ± 2.9 | | 3.5 |  |  |
|  | 10 | 91.3 ± 2.7 | 3.0 | 73.0 ± 4.9 | | 6.7 | 103.2 ± 9.6 | | 9.3 | 64.8 ± 11.8 | | 18.2 | **39.2 ± 14.2** | | **36.1** | 80.6 ± 26.0 | | **32.2** |  |  |
|  | 20 | 87.7 ± 5.0 | 5.8 | 68.8 ± 4.1 | | 5.9 | 104.3 ± 6.8 | | 6.5 | 64.5 ± 10.3 | | 15.9 | **48.0 ± 7.5** | | 15.7 | 70.4 ± 6.0 | | 8.5 |  |  |
| quercetin | 1.5625 | 79.5 ± 4.5 | 5.6 | 78.7 ± 2.8 | | 3.6 | 117.0 ± 9.9 | | 8.5 | 71.9 ± 7.9 | | 11.0 | **41.4 ± 2.5** | | 6.0 | 78.0 ± 7.1 | | 9.1 |  |  |
|  | 3.125 | 80.4 ± 3.0 | 3.7 | 71.9 ± 1.6 | | 2.2 | 105.2 ± 8.1 | | 7.7 | 75.7 ± 11.2 | | 14.8 | **23.4 ± 5.2** | | **22.1** | 71.1 ± 4.6 | | 6.5 |  |  |
|  | 6.25 | 85.3 ± 7.4 | 8.6 | 74.2 ± 3.5 | | 4.7 | 104.7 ± 10.8 | | 10.3 | 62.5 ± 7.9 | | 12.7 | **25.5 ± 2.0** | | 8.0 | 71.3 ± 10.6 | | 14.9 |  |  |
|  | 12.5 | 99.9 ± 7.9 | 7.9 | 78.4 ± 6.0 | | 7.7 | 115.6 ± 5.7 | | 4.9 | 93.8 ± 10.8 | | 11.5 | **30.3 ± 6.7** | | **22.0** | 74.6 ± 5.9 | | 7.8 |  |  |
| sodium  acetate | 70.25 | 89.5 ± 7.2 | 8.1 | 84.0 ± 4.6 | | 5.4 | 110.6 ± 6.6 | | 6.0 | 72.3 ± 17.5 | | **24.2** | 66.0 ± 4.2 | | 6.4 | 85.3 ± 9.2 | | 10.8 |  |  |
|  | 140.5 | 99.7 ± 10.4 | 10.4 | 83.6 ± 3.9 | | 4.7 | 111.5 ± 13.9 | | 12.5 | 71.9 ± 9.1 | | 12.7 | 56.0 ± 10.7 | | 19.1 | 81.3 ± 9.1 | | 11.2 |  |  |
|  | 281 | 117.4 ± 1.7 | 1.5 | 79.2 ± 4.9 | | 6.2 | 126.9 ± 6.4 | | 5.0 | 76.1 ± 13.4 | | 17.6 | 54.4 ± 8.3 | | 15.3 | 94.0 ± 9.4 | | 10.0 |  |  |
|  | 562 | 133.6 ± 11.1 | 8.3 | 83.8 ± 7.5 | | 9.0 | 141.8 ± 7.2 | | 5.1 | 91.4 ± 10.0 | | 10.9 | 58.1 ± 1.6 | | 2.7 | 123.4 ± 26.0 | | **21.0** |  |  |
| tannic acid | 2.5 | 119.2 ± 4.3 | 3.6 | 93.7 ± 7.3 | | 7.8 | 132.9 ± 7.2 | | 5.4 | 73.0 ± 15.7 | | **21.6** | **41.7 ± 2.6** | | 6.1 | 85.6 ± 12.2 | | 14.2 |  |  |
|  | 5 | 117.4 ± 2.4 | 2.1 | 84.1 ± 2.9 | | 3.4 | 149.8 ± 6.8 | | 4.5 | 69.3 ± 26.3 | | **38.0** | **31.2 ± 4.7** | | 15.1 | 75.3 ± 5.6 | | 7.5 |  |  |
|  | 10 | 99.3 ± 3.7 | 3.7 | 91.0 ± 8.7 | | 9.6 | 130.2 ± 16.8 | | 12.9 | 58.3 ± 25.9 | | **44.4** | **33.4 ± 5.3** | | 16.0 | **47.7 ± 3.9** | | 8.2 |  |  |
|  | 20 | 85.8 ± 5.9 | 6.8 | 103.2 ± 1.8 | | 1.8 | 100.6 ± 10.5 | | 10.4 | 57.8 ± 12.9 | | **22.3** | **36.3 ± 7.0** | | 19.2 | **44.5 ± 6.6** | | 14.9 |  |  |
| tannin | 25 | 65.6 ± 1.2 | 1.9 | 93.3 ± 4.3 | | 4.6 | 89.7 ± 3.0 | | 3.4 | **45.4 ± 6.4** | | 14.1 | **49.4 ± 7.4** | | 14.9 | 56.4 ± 8.7 | | 15.5 |  |  |
|  | 50 | 72.3 ± 4.6 | 6.3 | 93.3 ± 9.0 | | 9.7 | 94.2 ± 24.8 | | **26.3** | **49.9 ± 5.4** | | 10.7 | 72.2 ± 17.1 | | **23.7** | 56.1 ± 7.9 | | 14.0 |  |  |
|  | 100 | 65.3 ± 8.4 | 12.9 | 105.6 ± 8.4 | | 7.9 | 118.6 ± 27.8 | | **23.4** | **48.8 ± 7.8** | | 16.0 | 56.6 ± 8.1 | | 14.4 | 58.5 ± 4.7 | | 8.1 |  |  |
|  | 200 | 65.7 ± 7.8 | 11.9 | 78.8 ± 6.9 | | 8.7 | 113.1 ± 13.6 | | 12.0 | 64.3 ± 7.1 | | 11.1 | 90.1 ± 3.3 | | 3.6 | 101.5 ± 42.5 | | **41.9** |  |  |
| trypsin | 6.25 | 99.2 ± 5.2 | 5.3 | 101.6 ± 6.3 | | 6.2 | 103.3 ± 3.5 | | 3.4 | 88.3 ± 25.9 | | **29.4** | 67.9 ± 6.0 | | 8.8 | 88.6 ± 16.5 | | 18.6 |  |  |
|  | 12.5 | 108.7 ± 5.1 | 4.7 | 92.2 ± 2.9 | | 3.2 | 109.4 ± 4.6 | | 4.2 | 77.5 ± 19.6 | | **25.2** | 59.4 ± 8.0 | | 13.4 | 123.6 ± 36.6 | | **29.6** |  |  |
|  | 25 | 107.9 ± 9.2 | 8.5 | 92.3 ± 4.5 | | 4.8 | 109.1 ± 5.8 | | 5.3 | 73.9 ± 20.3 | | **27.5** | 53.6 ± 3.8 | | 7.1 | 94.7 ± 6.8 | | 7.1 |  |  |
|  | 50 | 117.1 ± 7.5 | 6.4 | 87.2 ± 3.6 | | 4.2 | 120.9 ± 8.2 | | 6.8 | 79.9 ± 18.8 | | **23.5** | **31.9 ± 6.8** | | **21.4** | 64.4 ± 3.5 | | 5.4 |  |  |

^a^Data was normalized to the maximal untreated control. Each assay (n=3) displayed separately. Expressed as average of each condition ± SD. Four replicates per condition in each assay. Bolded black indicates decrease of at least 50 %; red indicates increase above 150 %; blue indicates CV over 20 %

**Table S5** Medium signals in compound screening^a^ microplates produced by 32 µg/mL tetracycline

|  | Percentage of MAX (%) | |  |
| --- | --- | --- | --- |
| Plate | Resazurin assay | Crystal violet assay |  |
| 1 | 52.6 ± 8.1 | 62.3 ± 23.9 | |
| 2 | 54.4 ± 12.1 | 41.9 ± 11.4 | |
| 3 | 40.4 ± 4.2 | 74.2 ± 22.7 | |

^a^Normalized to untreated maximal bacterial growth (MAX). Results presented as average ± SD. The number of replicates was 8–16 wells. Samples contained 1 % DMSO

**Table S6** Data of minimum (MIN), medium (MID) and maximum (MAX) raw signals and their parameters in compound screening assays (n = 3). Technical replicates: 8 – 16 wells

| Resazurin | | | | | | | | | | | | | |
| --- | --- | --- | --- | --- | --- | --- | --- | --- | --- | --- | --- | --- | --- |
| Assay | DMSO (%) | AVG of MIN | AVG of MID | AVG of MAX | SD of MIN | SD of MID | SD of MAX | Z' | CV (%) of MIN | CV (%) of MID | CV (%) of MAX | S/N | S/B |
| 1 | 1 | 283.1 | 670.8 | 1020.7 | 4.3 | 59.8 | 21.3 | 0.9 | 1.5 | 8.9 | 2.1 | 172.9 | 3.6 |
|  | 0 | 286.7 | 709.9 | 1285.8 | 4.8 | 80.0 | 41.0 | 0.9 | 1.7 | 11.3 | 3.2 | 207.3 | 4.5 |
| 2 | 1 | 323.7 | 572.0 | 780.5 | 7.2 | 55.4 | 52.8 | 0.6 | 2.2 | 9.7 | 6.8 | 63.8 | 2.4 |
|  | 0 | 327.7 | 674.6 | 1015.6 | 5.3 | 120.2 | 52.0 | 0.7 | 1.6 | 17.8 | 5.1 | 128.8 | 3.1 |
| 3 | 1 | 232.5 | 549.3 | 1016.8 | 3.4 | 33.1 | 59.3 | 0.8 | 1.5 | 6.0 | 5.8 | 228.4 | 4.4 |
|  | 0 | 235.5 | 581.8 | 1324.8 | 4.4 | 182.2 | 113.5 | 0.7 | 1.9 | 31.3 | 8.6 | 248.9 | 5.6 |
| Crystal violet | | | | | | | | | | | | | |
| Assay | DMSO (%) | AVG of MIN | AVG of MID | AVG of MAX | SD of MIN | SD of MID | SD of MAX | Z' | CV (%) of MIN | CV (%) of MID | CV (%) of MAX | S/N | S/B |
| 1 | 1 | 0.1531 | 0.7949 | 1.1839 | 0.0167 | 0.2462 | 0.2908 | 0.1 | 10.9 | 31.0 | 24.6 | 61.9 | 7.7 |
|  | 0 | 0.1462 | 0.9607 | 1.5283 | 0.0156 | 0.3222 | 0.4422 | 0.0 | 10.7 | 33.5 | 28.9 | 88.4 | 10.5 |
| 2 | 1 | 0.1404 | 0.3920 | 0.7404 | 0.0196 | 0.0683 | 0.1358 | 0.2 | 14.0 | 17.4 | 18.3 | 30.6 | 5.3 |
|  | 0 | 0.1284 | 0.4761 | 0.7915 | 0.0149 | 0.1360 | 0.1119 | 0.4 | 11.6 | 28.6 | 14.1 | 44.5 | 6.2 |
| 3 | 1 | 0.1465 | 0.7771 | 0.9961 | 0.0141 | 0.1933 | 0.1681 | 0.4 | 9.6 | 24.9 | 16.9 | 60.2 | 6.8 |
|  | 0 | 0.1448 | 0.7140 | 1.1711 | 0.0154 | 0.2175 | 0.1926 | 0.4 | 10.7 | 30.5 | 16.4 | 66.6 | 8.1 |

## References

Cruz CD, Shah S, Tammela P (2018) Defining conditions for biofilm inhibition and eradication assays for Gram-positive clinical reference strains. BMC Microbiology 18(1):173 <https://doi.org/10.1186/s12866-018-1321-6>
